# Walking reduces sensorimotor network connectivity compared to standing

**DOI:** 10.1186/1743-0003-11-14

**Published:** 2014-02-13

**Authors:** Troy M Lau, Joseph T Gwin, Daniel P Ferris

**Affiliations:** 1 Human Neuromechanics Laboratory, School of Kinesiology University of Michigan, Ann Arbor, MI 48109-2214, USA; 2 US Army Research Laboratory, Human Research and Engineering Directorate, Translational Neuroscience Branch, Aberdeen Proving Ground, MD 21005, USA

**Keywords:** EEG (electroencephalography), Walking, Connectivity, Multi-tasking, Brain

## Abstract

**Background:**

Considerable effort has been devoted to mapping the functional and effective connectivity of the human brain, but these efforts have largely been limited to tasks involving stationary subjects. Recent advances with high-density electroencephalography (EEG) and Independent Components Analysis (ICA) have enabled study of electrocortical activity during human locomotion. The goal of this work was to measure the effective connectivity of cortical activity during human standing and walking.

**Methods:**

We recorded 248-channels of EEG as eight young healthy subjects stood and walked on a treadmill both while performing a visual oddball discrimination task and not performing the task. ICA parsed underlying electrocortical, electromyographic, and artifact sources from the EEG signals. Inverse source modeling methods and clustering algorithms localized posterior, anterior, prefrontal, left sensorimotor, and right sensorimotor clusters of electrocortical sources across subjects. We applied a directional measure of connectivity, conditional Granger causality, to determine the effective connectivity between electrocortical sources.

**Results:**

Connections involving sensorimotor clusters were weaker for walking than standing regardless of whether the subject was performing the simultaneous cognitive task or not. This finding supports the idea that cortical involvement during standing is greater than during walking, possibly because spinal neural networks play a greater role in locomotor control than standing control. Conversely, effective connectivity involving non-sensorimotor areas was stronger for walking than standing when subjects were engaged in the simultaneous cognitive task.

**Conclusions:**

Our results suggest that standing results in greater functional connectivity between sensorimotor cortical areas than walking does. Greater cognitive attention to standing posture than to walking control could be one interpretation of that finding. These techniques could be applied to clinical populations during gait to better investigate neural substrates involved in mobility disorders.

## Background

Analysis of functional and effective connectivity across distributed brain regions can provide new insight into how the brain functions. ‘Functional’ connectivity considers only the correlation between signals while ‘effective’ connectivity also maps the causal relationships between signals [1]. The brain contains a highly complex collection of neurons that interact and communicate in order to perform motor and cognitive actions. Many studies have examined human brain connectivity using functional Magnetic Resonance Imaging (fMRI) and Positron-Emission Tomography (PET) by treating image voxels as anatomical network nodes [2-6]. A drawback to these methods is that they require subjects to remain still during imaging, resulting in the study of constrained and somewhat artificial behaviors.

One way to study brain connectivity during more natural, whole body, behaviors is to combine high-density electroencephalography (EEG), Independent Component Analysis (ICA), and source localization techniques. We have recently demonstrated that high-density electroencephalography (EEG) combined with Independent Component Analysis (ICA) enables the study of electrocortical activity related to locomotor control and cognition during walking [7-9]. Clustering electrocortical sources across subjects according to spatial and spectral properties enables calculation of changes in effective connectivity between cortical regions using techniques such as Granger causality [10].

Previous studies have demonstrated that the cortex plays a significant role in postural control during standing [11,12] but there is much less information about cortical control of human walking. Walking relies heavily on spinal locomotor networks that are capable of generating rhythmic muscle activity [13-16]. While considerable evidence has documented the importance of spinal central pattern generators in non-human vertebrates, spinally generated locomotor activity in humans without functional descending motor pathways has proven to be difficult to document [13,17-19]. Transcranial magnetic stimulation [20-22] and functional near-infrared spectroscopy (fNIRS) [23-27] have been used to investigate cortical connectivity during locomotion. These approaches each have limitations in measurement area or temporal resolution that restrict their use to assess cortical network connectivity during gait. For example, fNIRS is limited to measuring activity in the outer cortex and the temporal resolution is limited to a few seconds at best.

The purpose of this paper was to assess the relative effective cortical connectivity in humans during walking and standing. We examined cortical connectivity in healthy young subjects under the following four conditions: walking while performing a simple cognitive task, walking without the concurrent task, standing while performing a simple cognitive task, and standing without the concurrent task. We hypothesized that the effective connectivity among independent sensorimotor electrocortical processes would be lower during walking than during standing. We based this hypothesis on the belief that standing in humans is predominantly controlled by supraspinal mechanisms and walking in human relies substantially on spinal neural networks.The inclusion of a concurrently performed cognitive task allowed us to examine whether these changes were consistent in the presence of competing attentional demands [28].

## Methods

### Experimental design

Eight healthy volunteers (7 males and 1 female) between the ages of 20–31 years participated in the study. None had any history of major lower limb injury or known neurological or locomotor deficits. All subjects were provided with, and signed, consent forms prior to the experiment. All procedures were approved by the University of Michigan Internal Review Board and complied with the standards defined in the Declaration of Helsinki. All processing and analysis was performed in Matlab (The Mathworks, Natick, MA) using scripts based on EEGLAB (sccn.ucsd.edu/eeglab), an open source environment for processing electrophysiological data [29] or the Granger Causality Toolbox [30].

Subjects stood (0.0 m/s) and walked (0.8 m/s and 1.25 m/s) on a treadmill while we recorded 248-channel electroencephalography at 512 Hz (ActiveTwo, BioSemi, Amsterdam, The Netherlands). Before data collection, the locations of the electrodes were measured with respect to anatomical head reference points and electrode gel was used to bring electrode impedance below 25 kΩ. Subjects participated in a visual odd-ball discrimination and response task for some of the data collection. Standard (80%) and target (20%) stimuli (vertical or 45° rotated black crosses on a white background, respectively) were displayed on a monitor placed at eye level about 1 m in front of the subjects. For each movement condition subjects completed one test block where they actively responded to the target stimuli by pressing a handheld trigger (we refer to this condition as *engaged*) and one test block where they passively observed the screen (we refer to this condition as *passive*). Each session began with the standing condition (5 minutes), followed by the walking conditions (10 minutes each) in random order.

Identical preprocessing steps were applied across all subjects and trials. Furthermore, data were appended for identical subjects across sessions to compute identical statistics and allow for identical IC/dipole comparison between sessions. This approach also allows for robust comparisons to be made between the session, as you will see in the following sections. After collection, EEG data were high-pass filtered above 1 Hz. 60 Hz line noise was also removed. As in [7,8], EEG signals exhibiting substantial noise throughout the collection were removed from the data in the following manner: 1) channels with std. dev. > 1000 μV were removed, 2) any channel whose kurtosis was more than 5 std. dev. from the mean was removed, and 3) channels that were uncorrelated (r < 0.4) with nearby channels for more than 1% of the time-samples were removed.

Prior to performing ICA decomposition, time periods of EEG with substantial artifact, as defined by z-transformed power across all channels, across the appended subject specific sessions, in a given 2 second time window being larger than 0.8, were rejected using EEGLAB. An average of 130.4 EEG channels were retained for analysis (Range - 89–164; STD - 24.6). We refer the reader to [7-9], for a more in depth overview of this approach. These remaining channel signals were then re-referenced to an average reference across the scalp and mastoid external channels. The remaining channels and epochs were input into to an adaptive mixture ICA algorithm [AMICA] [31,32] that utilizes the infomax [33] approach. ICA linearly decomposes EEG signals into a set of maximally independent components (ICs) [34].

### Data analysis

**Figure 1:**
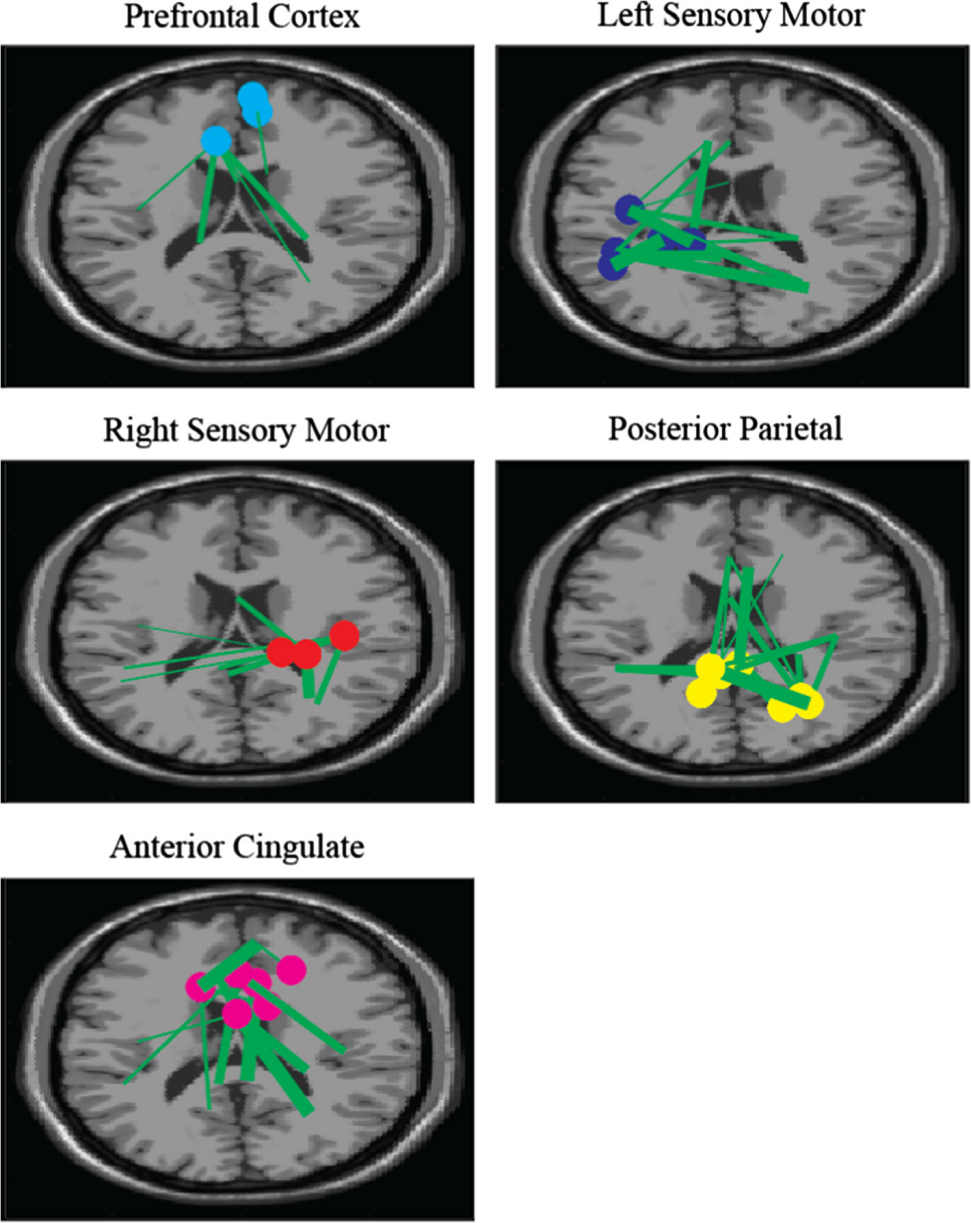
**Granger causality network connections for all subjects while walking at 1.25 m/s while not engaged in the visual oddball task.** Nodes shown are from statistically significant connections aggregated across all subjects. Nodes were in slightly different locations for each subject, some subjects had multiple nodes/region some had none. The thicker the line, the stronger the Granger Causality value. Nodes are clustered and color coated per their anatomical regions. Significant connections were determined by the F-statistic.

We then estimated an anatomical source location for each IC using DIPFIT functions within EEGLAB [35]. DIPFIT computes an equivalent current dipole model that best explains the scalp topography of each IC using a boundary element head model. ICs were excluded if the projection of the equivalent current dipole to the scalp accounted for less than 85% of the scalp map variance, or if the topography, time-course, and spectra of the IC were reflective of eye movement or electromyographic artifact [36,37]. The remaining ICs then reflected electrocortical sources. These sources were clustered across subjects using EEGLAB routines that implemented k-means clustering on vectors jointly coding differences in equivalent dipole locations and power spectra. Prior to clustering, the resulting joint vector was reduced to 10 principal dimensions using principal component analysis, as in (Gwin, Gramann et al. 2011), setting the maximal number of clusters. Clusters of electrocortical sources existed in prefrontal (PFC)(5 sources), anterior cingulate (AC) (9 sources), posterior parietal (PC) (13 sources), left sensorimotor (LSM) (7 sources), and right sensorimotor cortex (RSM) (6 sources) (Figure 1). The effective number of sources used may have been smaller depending on the significance/strength of connections coming from them.

We applied conditional Granger causality analysis to each subject’s electrocortical source signals to assess effective connectivity between the cortical regions. Granger causality is a directional connectivity measure that determines the linear causal influence of one signal on another [30,38,39] through a statistical test of the null hypothesis that a particular signal ‘Granger causes’ another signal. The postulate is that each signal is a linear combination of all other signals at a previous time (i.e., the order parameter). A full mathematical description of Granger causality has been published previously [39,40]. In short the linear regression is,

$$X_{i}\left(t\right)=\sum_{j=i}^{k}A_{\mathrm{ij}}X_{j}\left(t-\tau \right)+E\left(t-\tau \right)$$

where *X*_*i*_ is a signal at time *t, X*_*j*_ *= X*_*i…k*_ are all signals in the system/network, τ is the order parameter or time lag, and *E* is the residual. A signal is considered to ‘Granger cause’ another if, by adding the signal to the system, *E* is sufficiently reduced.

We calculated conditional temporal Granger causality values for all intra-subject electrocortical source signals with the aid of the freely distributed Granger Causality Toolbox [30]. To compute Granger causality, electrocortical source signals were divided into 10 second epochs before being demeaned and detrended. The best order parameter for each pair of electrocortical sources was then computed using the Bayesian information criterion and, on average, corresponded to about 20 ms, with a low of 15 ms and high of 25 ms. Varying the order parameter within this range, for all pairs, did not greatly affect the connectivity values. IC/dipole time series were checked for stationary using the Kwiatkowski-Phillips-Schmidt-Shin test and the Augmented Dickey Fuller test, and were not used in the connectivity analysis if they did not pass both. Granger causality values representing connectivity between electrocortical sources within the same brain region were ignored. Figure 1 demonstrates the anatomical locations of these regions, for all subjects, while walking at 1.25 m/s. For clarity only the most significant connections (determined by the F-statistic) are shown.

Average Granger causality values were computed for each intra-subject pair of electrocortical sources, within each condition, across the 10 second epochs. We evaluated the difference in connectivity strengths for each pair of electrocortical sources between walking and standing; cognitively engaged and cognitively passive conditions were treated separately. In addition, during standing we evaluated the difference in connectivity strengths for each pair of electrocortical sources between the cognitively engaged and cognitively passive conditions. Next, pairs of sources were grouped as *sensorimotor* if at least one of the sources in the pair was from the left or right sensorimotor cluster and *non-sensorimotor* otherwise. Connections coupling identical regions across subjects were then grouped and averaged. One-sample t-tests assessed whether the differences in connectivity strengths within each group (i.e., sensorimotor and non-sensorimotor) were significant; the p-value threshold was 0.05.

We also analyzed the Granger Causality differences using a second approach to ensure statistical validity. Instead of averaging similar region-region connections for each subject, then averaging across subjects, all sensorimotor and non-sensorimotor connections were binned into respective groups, and averages were taken for each. Then 10,000 surrogate data sets were created by taking the same connections (calculated over 10 second epochs) and randomly shuffling the regions they connected. The average connectivity for the surrogate sensorimotor regions and non-sensorimotor regions were then calculated as the statistical baseline, and the standard deviation of this surrogate distribution was used as our confidence level. For the sensorimotor regions there were an average of 10.5/16 connections per subject, and for the non-sensorimotor regions there were an average of 5.5/9 connections per subject.

## Results

**Figure 2:**
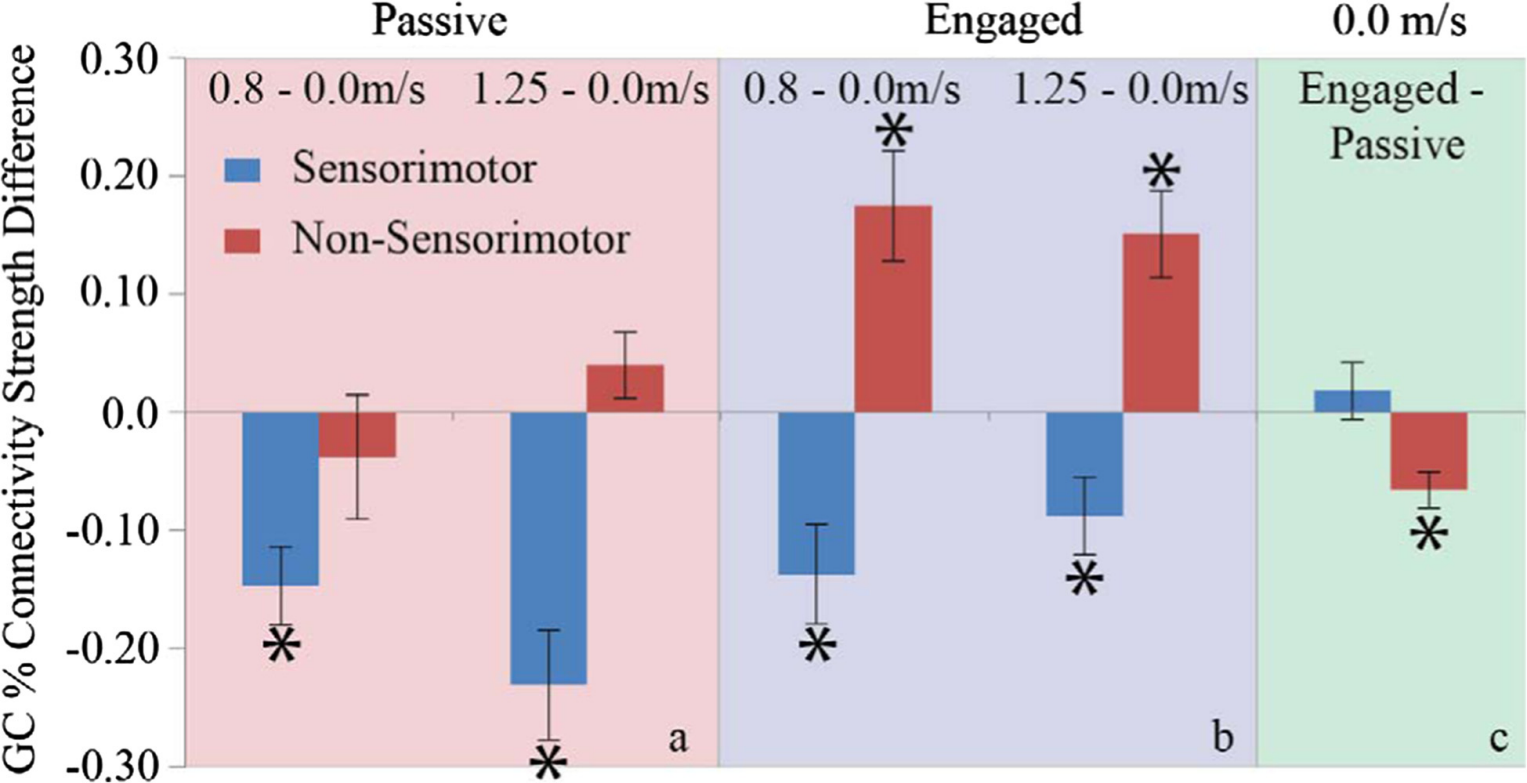
**Grand average percentage changes in network connectivity between conditions.** Connectivity during walking minus connectivity during standing is shown for sensorimotor (blue) and non-sensorimotor (red) network nodes when subjects were not engaged in the cognitive task (panel **a**) and when subjects were engaged in the cognitive task (panel **b**). Walking significant decreases sensorimotor network connectivity compared to standing and, when subjects are actively engaged in the cognitive task (panel **b**), walking significantly increase non-sensorimotor network connectivity. Panel **c** shows connectivity during active standing (actively engaged in the cognitive task) minus connectivity during passive (not engaged in the cognitive task). Passive standing elicited greater non-sensorimotor network connectivity than active standing, demonstrating the expected non-sensorimotor default mode network. * = p < 0.04.

The Granger causality analyses identified three main changes in connectivity among brain regions across conditions (Additional file 1: Table S1). First, connections involving the sensorimotor cortex (including both communication with association areas and interhemispheric communication within the sensorimotor cortex) were significantly weaker during walking than during standing. This was true regardless of whether the subject was actively engaged in the visual oddball discrimination and response task (p < 0.03) or the subject was passively observing the screen (p < 0.001) (Figures 2a & 2b, blue bars). Second, effective connectivities involving non-sensorimotor areas (i.e., prefrontal, posterior, and anterior clusters) were significantly stronger during walking than standing only when subjects were engaged in the simultaneous cognitive task (p < 0.03) (Figure 2b, red bars). Third, during standing, effective connectivities involving non-sensorimotor areas were significantly weaker when subjects were actively engaged in the visual oddball discrimination and response task than when subjects passively observed the visual stimuli (p < 0.02) (Figure 2c, red bars).

The second statistical analysis provided similar results to the primary. Some important results are stated here and all data are presented in Table 1. For the passive case subjects showed an average connectivity decrease of 13.38% in sensorimotor regions for walking at 0.8 m/s compared to standing. The surrogate sample produced an average decrease of -7.62 ± 4.91%. Similarly, a connectivity decrease of 20.64% was measured in sensorimotor regions for walking at 1.2 m/s compared to standing. The surrogate sample produced an average decrease of -9.57 ± 5.40%. For the active case subjects showed an average connectivity decrease of 9.31% in sensorimotor regions for walking at 0.8 m/s compared to standing. The surrogate sample produced an average increase of 3.87 ± 6.16%. Similarly, a connectivity decrease of 7.73% was measured in sensorimotor regions for walking at 1.2 m/s compared to standing. The surrogate sample produced an average increase of 2.06 ± 5.32%.

**Table 1:** Connectivity differences vs. random surrogate differences

**Sensorimotor differences (surrogate)**	**0.8 m/s – 0.0 m/s**	**1.2 m/s – 0.0 m/s**
Passive	-13.38% (-7.62 ± 4.91%)*	-20.64% (-9.57 ± 5.40%)*
Active	-9.31% (3.87 ± 6.16%)	-7.73% (2.06 ± 5.32%)
**Non-sensorimotor differences (surrogate)**	**0.8 m/s – 0.0 m/s**	**1.2 m/s – 0.0 m/s**
Passive	-0.52% (-8.09 ± 2.06%)	3.67% (-9.61 ± 2.24%)
Active	20.74% (4.09 ± 2.40%)*	15.63% (2.21 ± 2.11%)*

Each surrogate difference is calculated by averaging randomized connections (across all regions) over 1000 iterations. This is why the surrogate connectivities, in (), are the similar for both sensorimotor and non-sensorimotor regions. The variances are larger however, because the sample size (2 regions as opposed to 3) is smaller. Here statistical significance is indicated if any values deviate considerably from the surrogate average. We see that all results vary at least 1 STD from the surrogate mean (blue) and most deviate > 2 STD from the surrogate mean (green). * indicates as significant change from 0.

**Figure 3:**
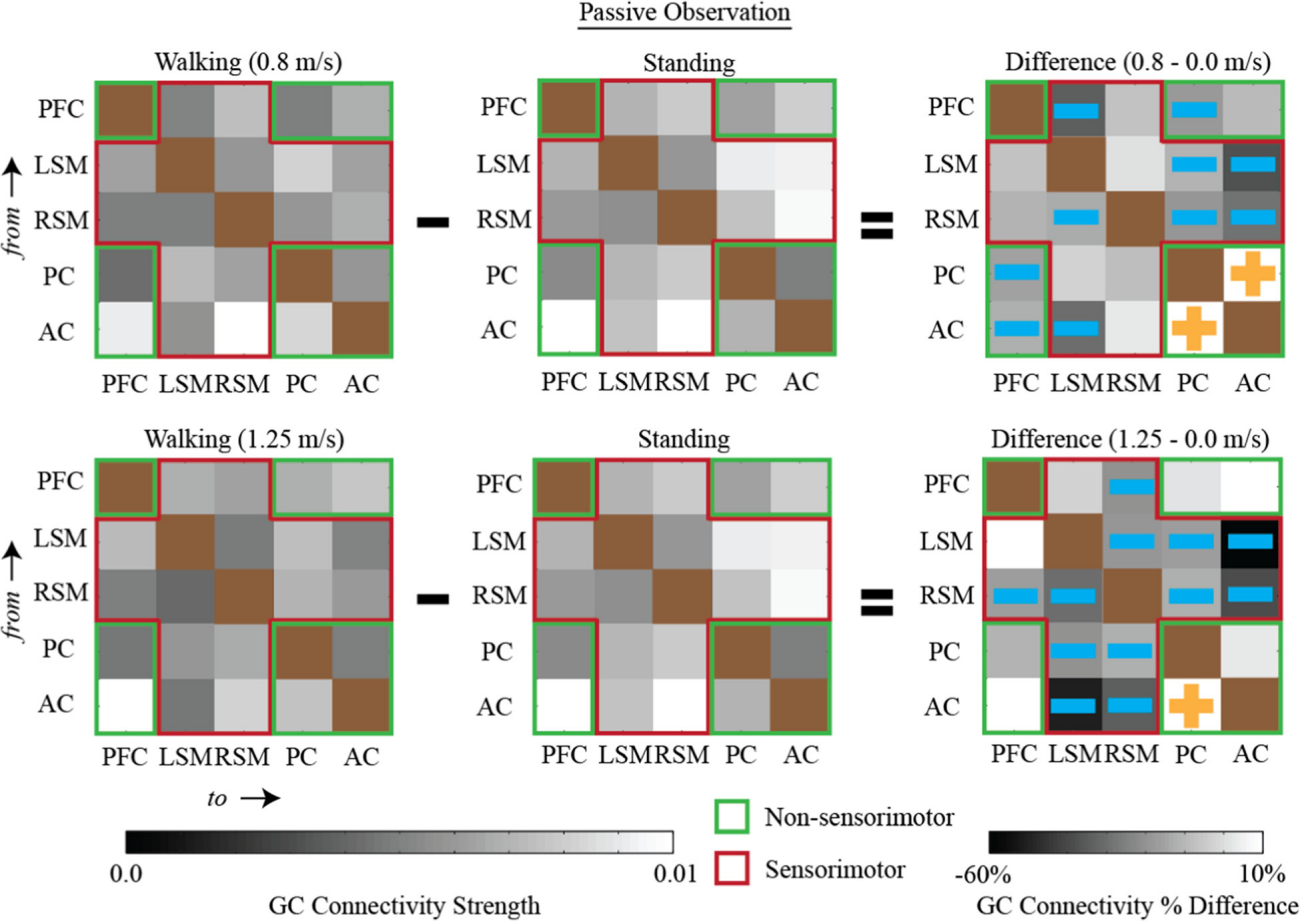
**Connectivity diagrams when subjects were not engaged in the cognitive task for (first column) walking, (second column) standing, and (third column) the connectivity strength differences between walking and standing (i.e., walking minus standing).** Walking speeds were (first row) 0.8 m/s and (second row) 1.25 m/s. The sensorimotor network nodes (left sensorimotor (LSM) and right sensorimotor (RSM)) are outlined in red and the non-sensorimotor network nodes (prefrontal cluster (PFC), posterior parietal cortex (PC), and anterior cingulate (AC)) are outlined in green. Statistically significant increases or decreases in connectivity strength that are more than two standard errors from zero are identified by gold plus signs and blue minus signs, respectively. All significant changes in connectivity strength between walking and standing within the sensorimotor network, at both speeds, were negative (i.e., functional connectivity involving sensorimotor areas was weaker during walking than during standing). The diagonal blocks are brown because no connectivity strengths were calculated for connections within the same brain area. GC Connectivity Strength can range from 0–1.

**Figure 4:**
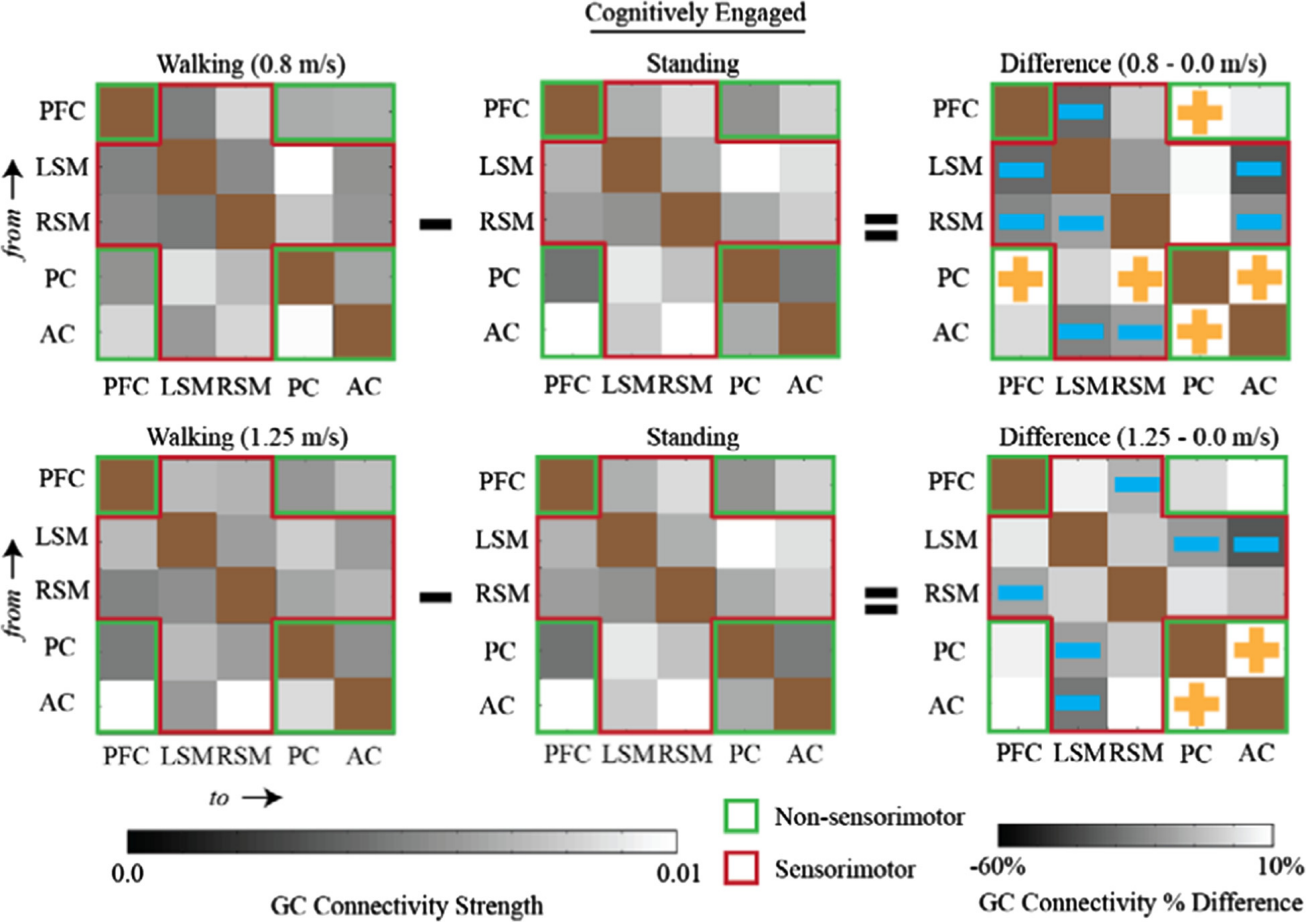
**Connectivity diagrams when subjects were actively engaged in the cognitive task for (first column) walking, (second column) standing, and (third column) the connectivity strength differences between walking and standing (i.e., walking minus standing).** Walking speeds were (first row) 0.8 m/s and (second row) 1.25 m/s. The sensorimotor network nodes (left sensorimotor (LSM) and right sensorimotor (RSM)) are outlined in red and the non-sensorimotor network nodes (prefrontal (PF), posterior parietal cortex (PC), and anterior cingulate (AC)) are outlined in green. Statistically significant increases or decreases in connectivity strength that are more than two standard errors from zero are identified by gold plus signs and blue minus signs, respectively. Nearly all significant changes in connectivity strength between walking and standing within the sensorimotor network, at both speeds, were negative (i.e., functional connectivity involving sensorimotor areas was weaker during walking than during standing). All significant changes in connectivity strength within the non-sensorimotor network, at both speeds, were positive (i.e., when actively engaged in a cognitive task, walking enhances connectivity among non-sensorimotor network nodes). The diagonal blocks are brown because no connectivity strengths were calculated for connections within the same brain area.

Matrix representations of directional network connectivity are shown in Figures 2, 3, and 4. In these figures, connections are *from* brain areas plotted vertically *to* brain areas plotted horizontally. The sensorimotor network nodes are outlined in red and the non-sensorimotor network nodes are outlined in green. Statistically significant differences in connection strength (more than 2 standard errors from 0) are indicated by gold plus signs (increases) and blue minus sings (decreases).

Regardless of whether or not subjects were actively engaged in the cognitive task, nearly all pairwise significant differences in connectivity strength, between walking at both speeds and standing, within the sensorimotor network, were negative (Figures 2 & 3). Connectivity involving sensorimotor areas was weaker during walking than during standing independent of the concurrent cognitive task. Note that Figure 3, which contains connectivity diagrams for standing and walking when subjects were not actively engaged in the cognitive task, shows the data used to calculate the grand averages shown in Figure 2a. Figure 4, which contains connectivity diagrams for when subjects were performing the visual oddball discrimination and response task, shows the data used to calculate the grand averages shown in Figure 2b.

When subjects were not actively engaged in the cognitive task, there were heterogeneous changes in the pairwise connectivity strength changes for the non-sensorimotor network. However, connectivity strengths between the PC and AC of electrocortical sources tended to be greater during walking than during standing (Figure 3). When subjects were actively engaged in the oddball discrimination task, all statistically significant (2 standard errors) differences in connectivity strength within the non-sensorimotor network, at both walking speeds, were positive (Figure 4). This indicates that walking uniformly enhanced connectivity among non-sensorimotor network nodes for the cognitive task condition.

**Figure 5:**
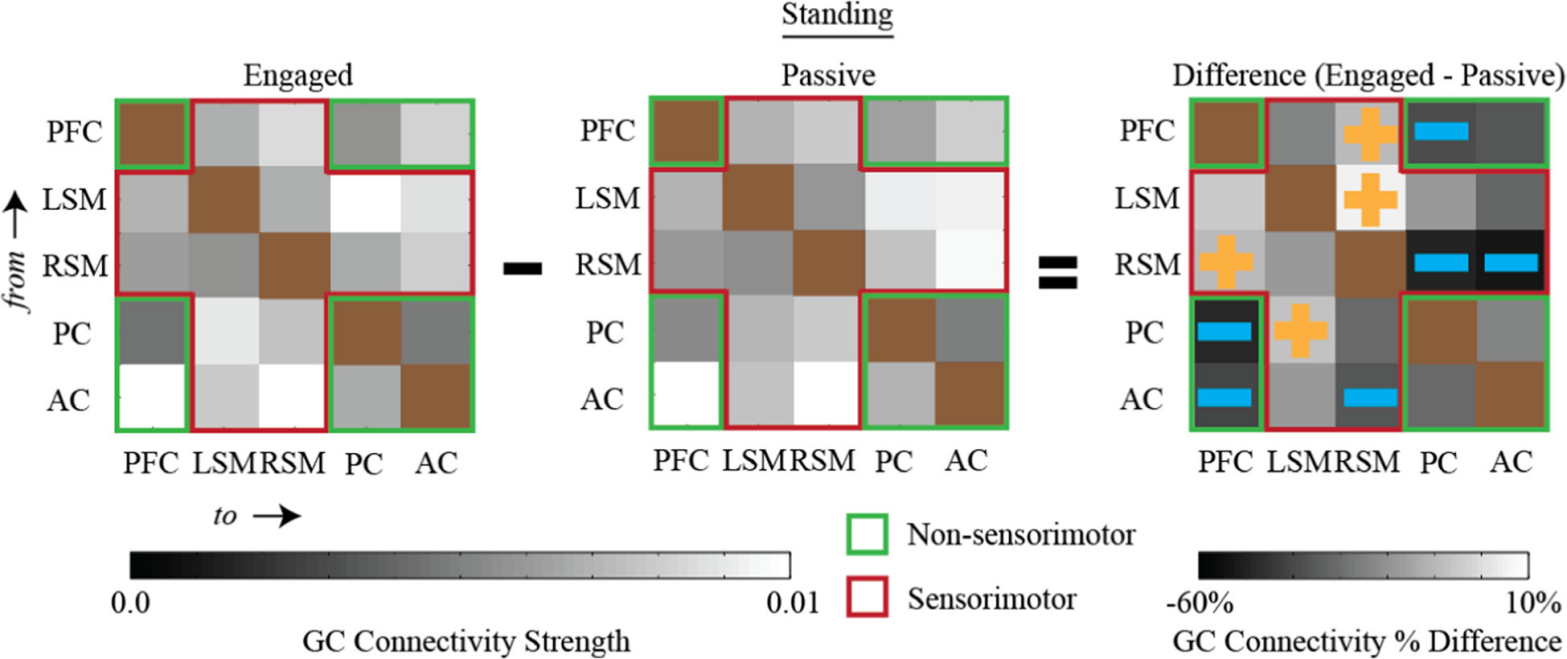
**Connectivity diagrams during standing (first column) when subjects were actively engaged in the cognitive task, (second column) when not actively engaged in the cognitive task, and (third column) the connectivity strength differences between active engagement and passive observation (i.e., cognitively active minus cognitively passive).** The sensorimotor network nodes (left sensorimotor (LSM) and right sensorimotor (RSM)) are outlined in red and the non-sensorimotor network nodes (prefrontal cortex(PFC), posterior parietal cortex (PC), and anterior cingulate (AC)) are outlined in green. Statistically significant increases or decreases in connectivity strength that are more than two standard errors from zero are identified by gold plus signs and blue minus signs, respectively. All significant changes in connectivity strength within the non-sensorimotor network were negative (i.e., the cognitive network was suppressed when subjects actively engaged in the cognitive task). The diagonal blocks are brown because no connectivity strengths were calculated for connections within the same brain area.

When comparing the no cognitive task standing to the cognitive task standing, all statistically significant differences in connectivity strength within the non-sensorimotor network were negative (Figure 5). Specifically, statistically significant weakening occurred for the prefrontal to/from PC cluster connections (bi-directionally) and for the AC to prefrontal cluster connection (uni-directionally). Note that Figure 5, which contains connectivity diagrams during standing with the cognitive task and without the cognitive task, shows the data used for the grand averages shown in Figure 2c.

## Discussion

We found a distinct, homogeneous, weakening in electrocortical sensorimotor network connectivity for walking compared to standing. While not all connectivity changed reached significance, all that did showed a sensorimotor weakening linked to walking (except for the PC-RSM conenction between 0.8 m/s and 0.0 m/s while cognitively engaged). Conversely, while engaged, the subjects showed a uniform increase in non-sensorimotor activity when walking. The most likely explanation for this finding is that standing requires considerable active cortical control for maintaining balance and posture [11,12], while walking relies more on spinal neural networks for producing the dominant muscle activation patterns. Given that locomotion requires less coordinated input from the brain than standing, it is not surprising that there is a measurable decrease in connectivity in sensorimotor networks during walking. Although there have been several studies using functional near-infrared spectroscopy to document changes in cortical activation during human locomotion [23-27,41] and during active balancing [42], there are no studies that have compared cortical activation during standing and walking. An important difference between the results presented here and previous studies analyzing cortical activation during walking with functional near-infrared spectroscopy is that our analyses focused on effective connectivity rather than just overall activity assessed through cerebral blood flow. The two approaches are not identical and are likely to result in some differences due to their measurements.

An important point to highlight is that there are many supraspinal neural substrates involved in the control of human locomotion other than just cortical areas. The brain stem, cerebellum, hippocampus, and basal ganglia also play a substantial role in the control of human walking and running [43-46]. Our study is limited in only being able to document cortical areas with spectral power synchronized with the gait cycle [7]. Future research that includes subject-specific head models for source localization and more advanced blind source separation algorithms might have more success in identifying other supraspinal sources involved in human locomotor control [47].

Another significant finding of this study was the increase in non-sensorimotor network connectivity strength during dual-task walking (i.e., walking while performing a cognitive task) compared to dual-task standing (i.e., standing while performing a cognitive task). This suggests that when engaged in a cognitive task, the act of walking increases the connectivity of the non-sensorimotor network while reducing the connectivity in the sensorimotor network. The data presented here do not allow us to probe the neurophysiological underpinnings of this observation. However, prior research leads to two possible interpretations. First, locomotion may enhance the performance and integration of brain regions associated with cognitive processing. Brisswalter et al. recently reviewed the evidence for acute exercise effects on cognitive performance and concluded that exercise improves non-motor performance [48]. Second, increased non-sensorimotor network connectivity may reflect the additional processing and communication needed for dual tasking. Walking at a controlled treadmill speed, where position on the treadmill must be continually monitored to prevent drifting off the belt, in addition to the cognitive demands of the oddball discrimination task may have required additional information processing in the non-sensorimotor network compared to standing while dual tasking [28].

In this study, we did not find consistent network differences between the two cognitive loading levels during walking. One possibility is that the connectivity effects of walking dominate over the effect of the simple cognitive task we employed. Another limitation of the study was the inability to assess the effect of walking speed given that only two walking speeds were evaluated. Future studies should examine a wider range of walking speeds and additional types of cognitive loading.

Unfortunately, our current approach does not allow for robust network analysis and graph theoretical approaches because of the limited number of nodes (electrocortical sources) that are extracted from EEG using ICA. If more electrocortical sources could be extracted then the non-sensorimotor network could likely be broken down into multiple other brain networks (e.g., default-mode and attentional networks). Additionally, integration across small and large time scales, as well as short and long distances, may provide valuable insights into the distributed processing of brain networks.

It is important to note the effect ICA has on the Granger causality measures. Although ICA extracts maximally independent signals, these signals are maximally simultaneously independent. On the other hand, Granger causality measures the contribution of one signal to another at time lags (1–20 ms in our case). Granger Causality is, therefore, well-suited for identifying causal relations between independent electrocortical source signals. In fact, Granger causality is susceptible to volume conduction in EEG measurements [49] but ICA can effectively remove instantaneous correlations due to volume conduction [50]. In contrast, the effects of the ICA transforms, coupled with other standard processing techniques like the removal of nearby uncorrelated channels may account for the overall low GC strengths (Figure 2) of ~ 0.01. It is likely that these techniques, along with the consideration of only a few specific anatomical regions, limits our computation of the complete causal network map.

It is also important to note that the amount of activity in a particular cortical region may not be correlated with the effective connectivity of that region. Therefore, increased effective connectivity does not necessarily indicate increased cortical activation. While previous cognitive studies show both increased network connectivity and increased overall activity [51-53], we have only demonstrated increased connectivity of the sensorimotor networks for standing compared to walking.

GC is also susceptible to detecting indirect connections between sources. Future work should investigate measures such as direct Directed Transfer Function (dDTF) and partial Directed Coherence (PDC). However the limited number of sources in this analysis would likely create extremely sparse networks. GC therefore is optimal for detecting closely coupled sources of activity (either directly or an open triplet). We believe that both cases are important for the experiment presented here.

## Conclusions

In summary, we found that effective sensorimotor connectivity was reduced during walking compared to standing, and that when humans are engaged in a cognitive task walking increased the effective connectivity of non-sensorimotor brain regions. These findings provide insight into how cortical regions interact during human gait and demonstrate the potential for future research studies to examine cortical connectivity in other mobile tasks. In particular, we have considerable optimism for applying these techniques to clinical populations with gait impairments such as ataxia and freezing gait.

## Abbreviations

ICA: Independent components analysis; IC: Independent component; EEG: Electroencephalography; GC: Granger causality; RSM: Right sensorimotor; LSM: Left sensorimotor; PFC: PreFrontal cluster (PFC); PC: Posterior cluster; AC: Anterior cluster.

## Competing interests

There are no competing interests in this work.

## Authors’ contributions

TL wrote the programming and performed all the analyses presented in this study. JG performed the experiments, collecting data on all subjects in this study. DF provided the inspiration for the experiment and provided useful feedback at all stages. All authors contributed to the writing of the manuscript and approved the final version.

## Supplementary Material

Additional file 1: Table S1 Individual subject region-region GC connectivity values. The individual connectivity values for each subject from region to region are presented for each condition. The * represents the lack of a connectivity value because a single node (or both nodes) did not exist for that subject over those regions. A superscript indicates the number of connections averaged for that value. While there are significant differences in baseline connectivity values across subjects, the changes across conditions are much more uniform. Click here for file

## References

[B1] Friston KJ Holmes AP Worsley KJ Poline JP Frith CD Frackowiak RSJ Statistical parametric maps in functional imaging: a general linear approach Hum Brain Mapp 1994 2 189 210 10.1002/hbm.460020402

[B2] Eguiluz VM Chialvo DR Cecchi GA Baliki M Apkarian AV Scale-free brain functional networks Phys Rev Lett 2005 94 018102 15698136 10.1103/PhysRevLett.94.018102

[B3] Greicius MD Krasnow B Reiss AL Menon V Functional connectivity in the resting brain: a network analysis of the default mode hypothesis Proc Natl Acad Sci U S A 2003 100 253 258 10.1073/pnas.0135058100 12506194 PMC140943

[B4] Meunier D Achard S Morcom A Bullmore E Age-related changes in modular organization of human brain functional networks Neuroimage 2009 44 715 723 10.1016/j.neuroimage.2008.09.062 19027073

[B5] Micheloyannis S Pachou E Stam CJ Vourkas M Erimaki S Tsirka V Using graph theoretical analysis of multi channel EEG to evaluate the neural efficiency hypothesis Neurosci Lett 2006 402 273 277 10.1016/j.neulet.2006.04.006 16678344

[B6] Stam CJ Nonlinear dynamical analysis of EEG and MEG: review of an emerging field Clin Neurophysiol 2005 116 2266 2301 10.1016/j.clinph.2005.06.011 16115797

[B7] Gwin JT Gramann K Makeig S Ferris DP Electrocortical activity is coupled to gait cycle phase during treadmill walking Neuroimage 2011 54 1289 1296 10.1016/j.neuroimage.2010.08.066 20832484

[B8] Gwin JT Gramann K Makeig S Ferris DP Removal of movement artifact from high-density EEG recorded during walking and running J Neurophysiol 2010 103 3526 3534 10.1152/jn.00105.2010 20410364 PMC3774587

[B9] Gramann K Gwin JT Bigdely-Shamlo N Ferris DP Makeig S Visual evoked responses during standing and walking Front Hum Neurosci 2011 4 202 21267424 10.3389/fnhum.2010.00202 PMC3024562

[B10] Ding M Chen Y Bressler SL 17 Granger causality: basic theory and application to neuroscience Handbook of time series analysis: recent theoretical developments and applications 2006 437

[B11] Tokuno CD Taube W Cresswell AG An enhanced level of motor cortical excitability during the control of human standing Acta Physiol 2009 195 385 395 10.1111/j.1748-1716.2008.01898.x 18774948

[B12] Vuillerme N Nafati G How attentional focus on body sway affects postural control during quiet standing Psychological Research 2007 71 192 200 10.1007/s00426-005-0018-2 16215747

[B13] Dietz V Colombo G Jensen L Baumgartner L Locomotor capacity of spinal cord in paraplegic patients Ann Neurol 1995 37 574 582 10.1002/ana.410370506 7755351

[B14] Dimitrijevic MR Gerasimenko Y Pinter MM Evidence for a spinal central pattern generator in humansa Ann N Y Acad Sci 1998 860 360 376 10.1111/j.1749-6632.1998.tb09062.x 9928325

[B15] Grillner S Neurobiological bases of rhythmic motor acts in vertebrates Science 1985 228 143 149 10.1126/science.3975635 3975635

[B16] Shik ML Orlovsky GN Neurophysiology of locomotor automatism Physiol Rev 1976 56 465 501 778867 10.1152/physrev.1976.56.3.465

[B17] Ferris DP Gordon KE Beres-Jones JA Harkema SJ Muscle activation during unilateral stepping occurs in the nonstepping limb of humans with clinically complete spinal cord injury Spinal Cord 2004 42 14 23 10.1038/sj.sc.3101542 14713939

[B18] Fong AJ Roy RR Ichiyama RM Lavrov I Courtine G Gerasimenko Y Tai YC Burdick J Edgerton VR Recovery of control of posture and locomotion after a spinal cord injury: solutions staring us in the face Progress in brain research 2009 175 393 418 19660669 10.1016/S0079-6123(09)17526-X PMC2904312

[B19] Wirz M Colombo G Dietz V Long term effects of locomotor training in spinal humans J Neurol Neurosurg Psychiatry 2001 71 93 96 10.1136/jnnp.71.1.93 11413270 PMC1737473

[B20] Schneider C Lavoie BA Capaday C On the origin of the soleus H-reflex modulation pattern during human walking and its task-dependent differences J Neurophysiol 2000 83 2881 2890 10805685 10.1152/jn.2000.83.5.2881

[B21] Iglesias C Nielsen JB Marchand-Pauvert V Corticospinal inhibition of transmission in propriospinal-like neurones during human walking Eur J Neurosci 2008 28 1351 1361 10.1111/j.1460-9568.2008.06414.x 18973562

[B22] Iglesias C Nielsen JB Marchand-Pauvert V Speed-related spinal excitation from ankle dorsiflexors to knee extensors during human walking Exp Brain Res 2008 188 101 110 10.1007/s00221-008-1344-6 18340438

[B23] Miyai I Tanabe HC Sase I Eda H Oda I Konishi I Tsunazawa Y Suzuki T Yanagida T Kubota K Cortical mapping of gait in humans: a near-infrared spectroscopic topography study NeuroImage 2001 14 1186 1192 10.1006/nimg.2001.0905 11697950

[B24] Suzuki M Miyai I Ono T Kubota K Activities in the frontal cortex and gait performance are modulated by preparation An fNIRS study NeuroImage 2008 39 600 607 10.1016/j.neuroimage.2007.08.044 17950626

[B25] Harada T Miyai I Suzuki M Kubota K Gait capacity affects cortical activation patterns related to speed control in the elderly Experiment brain resExperiment Hirnforsch Experiment cerebrale 2009 193 445 454 10.1007/s00221-008-1643-y 19030850

[B26] Kurz MJ Wilson TW Arpin DJ Stride-time variability and sensorimotor cortical activation during walking Neuroimage 2012 59 1602 1607 10.1016/j.neuroimage.2011.08.084 21920441

[B27] Suzuki M Miyai I Ono T Oda I Konishi I Kochiyama T Kubota K Prefrontal and premotor cortices are involved in adapting walking and running speed on the treadmill: an optical imaging study Neuroimage 2004 23 1020 1026 10.1016/j.neuroimage.2004.07.002 15528102

[B28] Yogev-Seligmann G Hausdorff JM Giladi N The role of executive function and attention in gait Mov Disord 2008 23 329 342 10.1002/mds.21720 18058946 PMC2535903

[B29] Delorme A Makeig S EEGLAB: an open source toolbox for analysis of single-trial EEG dynamics including independent component analysis J Neurosci Methods 2004 134 9 21 10.1016/j.jneumeth.2003.10.009 15102499

[B30] Seth AK A MATLAB toolbox for Granger causal connectivity analysis J Neurosci Methods 2010 186 262 273 10.1016/j.jneumeth.2009.11.020 19961876

[B31] Palmer JA Kreutz Delgado K Makeig S Rosca J, Erdogmus D, Principe JC, Haykin S Super Gaussian Mixture Source Model for ICA. In Lecture Notes in Computer Science 2006 Berlin: Springer 854 861

[B32] Palmer JA Makeig S Kreutz Delgado K Rao BD Newton Method for the ICA Mixture Model Book Newton Method for the ICA Mixture Model 2008 1805 1808 1805–1808

[B33] Bell AJ Sejnowski TJ An information-maximization approach to blind separation and blind deconvolution Neural Comput 1995 7 1129 1159 10.1162/neco.1995.7.6.1129 7584893

[B34] Makeig S Bell AJ Jung TP Sejnowski TJ Independent component analysis of electroencephalographic data Adv Neural Inf Process Syst 1996 8 145 151

[B35] Oostenveld R Oostendorp TF Validating the boundary element method for forward and inverse EEG computations in the presence of a hole in the skull Hum Brain Mapp 2002 17 179 192 10.1002/hbm.10061 12391571 PMC6872070

[B36] Jung TP Makeig S Humphries C Lee TW McKeown MJ Iragui V Sejnowski TJ Removing electroencephalographic artifacts by blind source separation Psychophysiology 2000 37 163 178 10.1016/S0167-8760(00)00088-X 10731767

[B37] Jung TP Makeig S Westerfield M Townsend J Courchesne E Sejnowski TJ Removal of eye activity artifacts from visual event-related potentials in normal and clinical subjects Clin Neurophysiol 2000 111 1745 1758 10.1016/S1388-2457(00)00386-2 11018488

[B38] Bressler SL Seth AK Wiener-Granger causality: a well established methodology Neuroimage 2011 In Press, Corrected Proof 10.1016/j.neuroimage.2010.02.059 20202481

[B39] Granger CWJ Investigating causal relations by econometric models and cross-spectral methods Econometrica 1969 37 424 438 10.2307/1912791

[B40] Geweke J Measurement of linear dependence and feedback between multiple time series J Am Stat Assoc 1982 77 304 313 10.1080/01621459.1982.10477803

[B41] Hatakenaka M Miyai I Mihara M Sakoda S Kubota K Frontal regions involved in learning of motor skillâ€”A functional NIRS study NeuroImage 2007 34 109 116 10.1016/j.neuroimage.2006.08.014 17067821

[B42] Karim H Schmidt B Dart D Beluk N Huppert T Functional near-infrared spectroscopy (fNIRS) of brain function during active balancing using a video game system Gait & posture 2012 35 3 367 372 10.1016/j.gaitpost.2011.10.007 22078300 PMC3294084

[B43] Jahn K Deutschlander A Stephan T Kalla R Wiesmann M Strupp M Brandt T Imaging human supraspinal locomotor centers in brainstem and cerebellum Neuroimage 2008 39 786 792 10.1016/j.neuroimage.2007.09.047 18029199

[B44] Jahn K Deutschlander A Stephan T Strupp M Wiesmann M Brandt T Brain activation patterns during imagined stance and locomotion in functional magnetic resonance imaging Neuroimage 2004 22 1722 1731 10.1016/j.neuroimage.2004.05.017 15275928

[B45] Jahn K Wagner J Deutschlander A Kalla R Hufner K Stephan T Strupp M Brandt T Human hippocampal activation during stance and locomotion: fMRI study on healthy, blind, and vestibular-loss subjects Ann N Y Acad Sci 2009 1164 229 235 10.1111/j.1749-6632.2009.03770.x 19645904

[B46] la Fougere C Zwergal A Rominger A Forster S Fesl G Dieterich M Brandt T Strupp M Bartenstein P Jahn K Real versus imagined locomotion: a [18 F]-FDG PET-fMRI comparison Neuroimage 2010 50 1589 1598 10.1016/j.neuroimage.2009.12.060 20034578

[B47] Delorme A Mullen T Kothe C Akalin Acar Z Bigdely Shamlo N Vankov A Makeig S EEGLAB, SIFT, NFT, BCILAB, and ERICA: new tools for advanced EEG processing Comput Intell Neurosci 2011 2011 130714 21687590 10.1155/2011/130714 PMC3114412

[B48] Brisswalter J Collardeau M Ren, eacute A Effects of acute physical exercise characteristics on cognitive performance Sports Med 2002 32 555 566 10.2165/00007256-200232090-00002 12096929

[B49] Haufe S Nikulin V Nolte G Identifying brain effective connectivity patterns from EEG: performance of Granger causality, DTF, PDC and PSI on simulated data BMC Neuroscience 2011 12 Suppl 1 P141 10.1186/1471-2202-12-S1-P141

[B50] Delorme A Palmer J Onton J Oostenveld R Makeig S Independent EEG sources are dipolar PLoS ONE 2012 7 e30135 10.1371/journal.pone.0030135 22355308 PMC3280242

[B51] Raichle ME MacLeod AM Snyder AZ Powers WJ Gusnard DA Shulman GL A default mode of brain function Proc Natl Acad Sci 2001 98 676 682 10.1073/pnas.98.2.676 11209064 PMC14647

[B52] Raichle ME Snyder AZ A default mode of brain function: a brief history of an evolving idea Neuroimage 2007 37 1083 1090 10.1016/j.neuroimage.2007.02.041 17719799

[B53] Uddin LQ Clare Kelly AM Biswal BB Xavier Castellanos F Milham MP Functional connectivity of default mode network components: correlation, anticorrelation, and causality Hum Brain Mapp 2009 30 625 637 10.1002/hbm.20531 18219617 PMC3654104

