# Supplementary material for: Walking reduces sensorimotor network connectivity compared to standing
Source: J Neuroeng Rehabil. 2014 Feb 13;11:14. doi: 10.1186/1743-0003-11-14 (PMC3929753; doi:10.1186/1743-0003-11-14)
Supplement: Additional file 1: Table S1 — Individual subject region-region GC connectivity values. The individual connectivity values for each subject from region to region are presented for each condition. The * represents the lack of a connectivity value because a single node (or both nodes) did not exist for that subject over those regions. A superscript indicates the number of connections averaged for that value. While there are significant differences in baseline connectivity values across subjects, the changes across conditions are much more uniform. [file 1743-0003-11-14-S1.doc]

Additional file 1: Table S1. Individual subject region-region GC connectivity values. The individual connectivity values for each subject from region to region are presented for each condition. The * represents the lack of a connectivity value because a single node (or both nodes) did not exist for that subject over those regions. A superscript indicates the number of connections averaged for that value. While there are significant differences in baseline connectivity values across subjects, the changes across conditions are much more uniform.

| **0.0 m/s** |  |  |  |  |  |
| --- | --- | --- | --- | --- | --- |
| **10^-3^** | **PFC** | **LSM** | **RSM** | **PC** | **AC** |
| **PFC** |  | *, 7.7^2^, 4.7^2^, *, *, *, *, 9.8^1^ | *, 14.5^1^, 2.9^1^, 11.9^1^, *, *, *, 5.0^2^ | *, 4.8^1^, *, 7.6^4^, *, 5.3^1^, *, 3.5^1^ | *, 12.7^1^, *, *, *, 4.5^3^, *, 14.2^1^ |
| **LSM** | *, 9.2^2^, 3.7^2^, *, *, *, *, 8.8^1^ |  | 3.5^1^, 9.0^2^, 2.6^2^, *, *, *, *, 7.0^2^ | 7.4^3^, 9.6^2^, *, *, 11.3^1^, *, *, 11.3^1^ | 4.4^1^, 8.8^2^, *, *, *, *, *, 15.3^1^ |
| **RSM** | *, 5.6^1^, 2.0^1^, 11.2^1^, *, *, *, 5.6^2^ | 3.3^1^, 6.1^2^, 3.0^2^, *, *, *, *, 8.7^2^ |  | 5.8^3^, 20.3^1^, *, 7.9^4^, *, *, *, 3.2^2^ | 4.6^1^, 15.8^1^, *, *, *, *, *, 9.02 |
| **PC** | *, 4.2^1^, *, 7.0^4^, *, 2.7^1^, *, 2.9^1^ | 6.4^3^,4.8^2^, *, *, 13.2^1^, *, *, 7.4^1^ | 5.2^3^, 13.0^1^, *, 10.9^4^, *, *, *, 3.0^2^ |  | 3.9^3^, 9.6^1^, *, *, *, 3.6^3^, 6.3^2^, 4.9^1^ |
| **AC** | *, 22.4^1^, *, *, *, 5.7^3^, *, 14.6^1^ | 5.2^1^,7.1^2^, *, *, *, *, *, 10.9^1^ | 7.5^1^, 20.8^1^, *, *, *, *, *, 8.1^2^ | 4.9^3^, 22.0^1^, *, *, *, 4.7^3^, 7.0^2^, 4.1^1^ |  |
| **0.8m/s** |  |  |  |  |  |
| **PFC** |  | *, 5.4^2^, 3.2^2^, *, *, *, *, 8.1^1^ | *, 12.6^1^, 2.1^1^, 11.6^1^, *, *, *, 5.5^2^ | *, 3.9^1^, *, 5.2^4^, *, 5.2^1^, *, 4.7^1^ | *, 7.3^1^, *, *, *, 4.1^3^, *, 14.0^1^ |
| **LSM** | *, 8.4^2^, 3.6^2^, *, *, *, *, 7.7^1^ |  | 2.6^1^, 8.3^2^, 3.2^2^, *, *, *, *, 7.7^2^ | 6.5^3^, 8.5^2^, *, *, 11.0^1^, *, *, 9.4^1^ | 4.2^1^, 4.9^2^, *, *, *, *, *, 11.1^1^ |
| **RSM** | *, 5.0^1^, 2.1^1^, 5.3^1^, *, *, *, 6.1^2^ | 2.6^1^, 4.2^2^, 2.7^2^, *, *, *, *, 9.1^2^ |  | 4.2^3^, 17.0^1^, *, 5.4^4^, *, *, *, 4.0^2^ | 4.0^1^, 10.4^1^, *, *, *, *, *, 6.4^2^ |
| **PC** | *, 3.0^1^, *, 4.7^4^, *, 3.7^1^, *, 4.3^1^ | 5.8^3^,4.1_2_, *, *, 16.8^1^, *, *, 9.0^1^ | 4.7^3^, 8.9^1^, *, 8.0^4^, *, *, *, 4.0+ |  | 3.8^3^, 7.7^1^, *, *, *, 3.5^3^, 10.8^2^, 7.9^1^ |
| **AC** | *, 21.5^1^, *, *, *, 4.6^3^, *, 10.7^1^ | 5.1^1^,4.4^2^, *, *, *, *, *, 8.6^1^ | 8.9^1^, 17.8^1^, *, *, *, *, *, 8.1^2^ | 5.4^3^, 20.6^1^, *, *, *, 4.6^3^, 12.7^2^, 7.5^1^ |  |
| **1.25m/s** |  |  |  |  |  |
| **PFC** |  | *, 7.9^2^, 5.5^2^, *, *, *, *, 6.9^1^ | *, 11.2^1^, 2.3^1^, 9.3^1^, *, *, *, 4.5^2^ | *, 4.2^1^, *, 6.9^4^, *, 12.9^1^, *, 2.7^1^ | *, 7.1^1^, *, *, *, 7.0^3^, *, 10.7^1^ |
| **LSM** | *, 8.5^2^, 6.1^2^, *, *, *, *, 6.9^1^ |  | 2.7^1^, 7.2^2^, 2.6^2^, *, *, *, *, 5.4^2^ | 7.0^3^, 7.9^2^, *, *, 11.0^1^, *, *, 8.4^1^ | 3.1^1^, 3.7^2^, *, *, *, *, *, 9.6^1^ |
| **RSM** | *, 3.8^1^, 2.3^1^, 7.8^1^, *, *, *, 5.3^2^ | 2.8^1^, 3.8^2^, 2.7^2^, *, *, *, *, 6.7^2^ |  | 4.3^3^, 18.0^1^, *, 8.4^4^, *, *, *, 2.2^2^ | 5.6^1^, 8.9^1^, *, *, *, *, *, 4.7^2^ |
| **PC** | *, 2.8^1^, *, 5.3^4^, *, 6.7^1^, *, 1.9^1^ | 5.1^3^,3.6^2^, *, *, 11.5^1^, *, *, 7.3^1^ | 4.1^3^, 10.3^1^, *, 9.8^4^, *, *, *, 2.3^2^ |  | 4.1^3^, 10.5^1^, *, *, *, 3.3^3^, 6.5^2^, 4.2^1^ |
| **AC** | *, 12.2^1^, *, *, *, 9.7^3^, *, 9.1^1^ | 3.2^1^,4.0^2^, *, *, *, *, *, 7.1^1^ | 7.3^1^, 16.6^1^, *, *, *, *, *, 4.5^2^ | 5.8^3^, 23.3^1^, *, *, *, 4.3^3^, 9.1^2^, 4.2^1^ |  |
